# To heal or not to heal: the ACL dilemma

**DOI:** 10.1186/s10195-020-00554-8

**Published:** 2020-08-29

**Authors:** Andrea Ferretti

**Affiliations:** Orthopaedic Unit and Kirk Kilgour Sports Injury Centre, S. Andrea Hospital, University of Rome La Sapienza, Rome, Italy

Anterior cruciate ligament (ACL) reconstruction represents one of the most commonly performed surgical procedures worldwide, with very good results in terms of restoring knee stability, recovery of function, and return to sports at a preinjury level [1].

Although substantial differences still exist among surgeons regarding the type of reconstruction (autologous, homologous, or allografts) and graft choice in case of autologous grafting (hamstrings, patellar tendon, or quadriceps tendon), there is general agreement that, when surgery is needed, ACL reconstruction is the gold standard [2].

However, in the last few years, the number of papers on direct repair of ACL published in international literature has increased, witnessing growing interest in this topic [3–7]. It appears that ACL repair should be reconsidered as an alternative to reconstruction, taking advantage of new surgical materials, devices, and techniques.

In fact, ACL repair was the first procedure ever reported in the history of ACL surgery, although the results of this pioneering surgery were never trustworthily reported [8–11]. Since the beginning of modern ACL surgery, dating back to the 1960s, results of ACL repair, as reported by several authors over the years, were overall unsatisfactory, leading knee surgeons to the conclusion that a torn ACL was irreparable.

However, deep study of the biological basis regulating the different phases of healing of human ligaments reveals that even the ACL seems to have potential to heal [12]. The blood supply, whose anatomy was deeply investigated by an Italian researcher, is rich in vessels and anastomosis, providing adequate supply to all kinds and sites of tears (proximal, distal, and midsubstance) [13, 14]. The early platelet clot seems to be able to deliver growth factors that attract mesenchymal cells, as well as governing their differentiation towards fibroblasts and myofibroblasts. In fact, these cells are found in large numbers in the stumps of a torn ACL, even months after an injury, as well as type 3 collagen, a precursor of the eventual ligamentous type 1 collagen [15].

Based on these factors, why is the ACL still somehow considered irreparable by so many surgeons? The answer could be anatomical and mechanical rather than biological. Lack of healing could be related to the intraarticular environment of this ligament, which could avoid formation of a suitable provisional bridge between the disrupted stumps [16]. Gravity would cause the distal stump to bend towards the posterior cruciate ligament (PCL), where it would eventually attach, taking advantage of the PCL’s rich synovial blood supply. Attachment of the ACL to the PCL results in a viable but nonfunctional ligament [15].

Should this be the case, surgery could actually be able to help Nature complete this healing process by reestablishing the continuity and tension of the ligament and promoting scar tissue formation and differentiation.

Returning to recently published literature on ACL repair, we notice that reported results are highly conflicting, with acceptable outcomes ranging from 40% to up to 90%. [17–20]. This great variability could be explained by the great variety of indications, surgical techniques, materials, and rehabilitation protocols.

This issue of *JOT* includes a paper written in collaboration with the Hospital for Special Surgery, presenting preliminary results of a technique for ACL repair evaluated by sequential magnetic resonance imaging (MRI). Inspection of some of the quite astonishing images shows that the presented technique, if used in select cases of acute ACL tears, could lead to encouraging results.

However, even if ACL repair may appear to be an attractive procedure to minimize morbidity and avoid harvesting-related complications, all preliminary outcomes should be taken very cautiously. Moreover, the correct indication in terms of timing of surgery (acute, subacute, and chronic), type of tears (proximal, distal, and midsubstance), age, sex, activity level, associated injuries, severity of instability, surgical technique, use of augmentation, etc. must be further addressed and possibly elucidated.

It seems easily predictable that, in the near future, other centers and surgeons will deal with this new challenging topic of ACL surgery and that the issue of “to repair or not repair” will become one of the hot topics at all future meetings and congresses; more submissions are expected from several authors to be considered for publication in all major specialized journals.

## Data Availability

Not applicable

## Abbreviations

ACL: Anterior cruciate ligament
MRI: Magnetic resonance imaging
PCL: Posterior cruciate ligament
